# Dispensing Oral Temozolomide in Children: Precision and Stability of a Novel and Ready to Use Liquid Formulation in Comparison with Capsule Derived Mixtures

**DOI:** 10.3390/pharmaceutics15122711

**Published:** 2023-11-30

**Authors:** Caroline Lemarchand, Hugues Bienaymé, André Rieutord, Samuel Abbou, Maxime Annereau, Jeremy Bastid

**Affiliations:** 1 ORPHELIA Pharma, 75005 Paris, France; hugues.bienayme@orphelia-pharma.eu; 2 Clinical Pharmacy Department, Gustave Roussy Cancer Campus, 94805 Villejuif, France; andre.rieutord@gustaveroussy.fr (A.R.); maxime.annereau@gustaveroussy.fr (M.A.); 3 Children and Adolescent Oncology Department, INSERM U1015, Paris-Saclay University, 94805 Villejuif, France; samuel.abbou@gustaveroussy.fr

**Keywords:** cancer, oral administration, solution, stability, temozolomide

## Abstract

Temozolomide (TMZ) is part of the therapeutic armamentarium used in managing pediatric cancers; however, available oral forms (capsules) are not adapted for use in children. Our aim was to assess the dose accuracy and stability of TMZ using capsule contents mixed with food compared with a novel, ready-to-use liquid formulation specifically developed for children (Ped-TMZ, brand name KIZFIZO). Dose accuracy and TMZ stability testing were performed with TMZ capsule contents (90 mg) mixed with food vehicles (apple juice, apple sauce, cream, milk, and mashed potatoes) and compared to an equivalent dose of Ped-TMZ. Acceptance criteria were predefined for TMZ (95.0–105.0%) and its degradation product amino-imidazole-carboxamide (AIC; <1%) content. The delivered dose was significantly higher using Ped-TMZ (96.6 ± 1.2%) and within the predefined criteria for TMZ content, whereas it was systematically under the lower specifications of 95% using capsule-derived preparations with apple juice (91.0 ± 1.5%) and apple sauce (91.6 ± 1.4%), respectively (*p* < 0.0001). In chemical stability tests, the four food vehicles (apple sauce, cream, milk, mashed potatoes) had a significant effect on TMZ stability (*p* = 0.0042), and the AIC significantly increased with time in three of the four vehicles (*p* < 0.0001). Only 1/72 of preparations from capsules met the predefined acceptance criteria, whereas Ped-TMZ showed no TMZ loss, and the AIC remained within specifications. In conclusion, mixing TMZ capsule content with food may result in significant underexposure, possibly even greater in routine practice, as complete food intake by the child is unlikely.

## 1. Introduction

Temozolomide (TMZ) is an alkylating agent with demonstrated schedule-dependent clinical activity against malignant gliomas such as glioblastoma multiforme or anaplastic astrocytoma in patients from three years of age [1,2]. TMZ is also widely used as standard chemotherapy to treat pediatric cancers, including neuroblastoma [3,4,5], medulloblastoma [6], and rhabdomyosarcoma [7]. TMZ development was driven by the increasing interest in the delivery of anticancer drugs via the oral route, which is more convenient for self-administration and improves patients’ quality of life. Solid dosage forms, i.e., tablets and capsules, are the most preferred pharmaceutical forms because of their advantages, such as high dosing accuracy, easy handling, long-term stability in ambient conditions, and patient compliance. As TMZ dosing is based on the body surface area (BSA), the drug is currently supplied as hard capsules of six different strengths (Temodal^®^: 5 mg, 20 mg, 100 mg, 140 mg, 180 mg, and 250 mg) to enable dosing flexibility [2].

Temozolomide demonstrated rapid (T_max_ ~1 h) and complete absorption (100% bioavailability) and a linear-to-dose pharmacokinetic (i.e., total body clearance) after oral administration of capsules in adult patients under fasting conditions [1,2]. However, the food effect specifically studied in a pharmacokinetic study in adult patients showed a significant impact on the pharmacokinetic profile with a T_max_ increase and C_max_ and AUC_0–24_ decrease [1]. As it cannot be excluded that the change in C_max_ is clinically significant, current product guidance recommends that TMZ capsules must be swallowed whole with water on an empty stomach [2]. Nevertheless, the TMZ capsules are large (from approximately 16 mm (size 3 for TMZ 5 mg) to 22 mm (size 0 for TMZ 140 mg to 250 mg) in length), making it difficult for pediatric patients to swallow [8]. Even if the age at which children can swallow intact tablets or capsules is highly dependent on the individual and the training that they receive from healthcare professionals or caregivers, the size of tablets and capsules should be kept as small as possible [9] and less than 10 mm [10] to improve the acceptability. Furthermore, the administration of several capsules may be required to achieve the prescribed dose, which represents an additional barrier for children who are averse to swallowing the solid dosage form [11]. For TMZ treatment, according to the available dosage strengths, the common dose of 90 mg (corresponding to a child under the age of 6 exhibiting a BSA of 0.6 m^2^ and treated with 150 mg/m^2^ of TMZ) is achieved with four capsules of 20 mg and two capsules of 5 mg, i.e., six capsules of size 2 or 3 to be swallowed by the child. The combination of size and number of hard capsules of TMZ can affect oral acceptability and potential adherence to chemotherapy treatment.

In these cases, clinicians may often instruct caregivers to follow common administration practices in pediatrics [12,13] by opening the TMZ capsules and mixing the powder content into liquid and/or soft food before spoon-feeding or syringing the mixture to the child with the aim of improving swallowability and palatability [14,15]. Despite the lack of scientific information regarding the adverse effects associated with such drug manipulations or modifications [16], mixing medicines into foodstuffs to facilitate drug administration is frequent and occurs in one-third of medicines for oral administration to the pediatric population [11,17]. However, unless the impact of liquid or soft foods used as vehicles for drug administration on the drug product performance (i.e., drug product quality attributes) is appropriately assessed and justified [18], this approach should be avoided for several reasons. Firstly, TMZ is a cytotoxic and teratogenic alkylating agent, meaning that exposure to the powder can be harmful to caregivers and the family [8,15]. Secondly, administering the accurate dose may be inconsistent and compromised by incomplete food consumption by the child. Indeed, TMZ is a bitter substance and drug loss spilled, regurgitated, or left behind in the glass/pot is frequent. Manipulation and opening of the capsules may further increase the loss of TMZ (e.g., some powder left in the capsule shell) and thereby increase errors in accurate dosing. Third, as TMZ is unstable under light and at a pH of 7 or above, mixing the capsule contents with food may result in drug degradation and suboptimal dosing [15]. Finally, the co-administration of capsules with food may impact the bioavailability of TMZ [1] with the potential risk of subtherapeutic drug levels.

Considering these challenges, the European Medicines Agency (EMA) ‘Draft Inventory of Paediatric Therapeutic Needs’ highlighted the need for an age-appropriate formulation of TMZ [19].

Ped-TMZ (KIZFIZO^®^, temozolomide 40 mg/mL oral suspension) is a ready-to-use oral liquid pediatric formulation of TMZ that is currently in development for the treatment of relapsed or refractory neuroblastoma. This age-adapted and taste-masked formulation is designed to deliver an accurate high drug load while avoiding drug manipulation and caregiver exposure to TMZ. The objective of this study was to evaluate the impact of TMZ capsule manipulation on the main critical quality attributes, accuracy (assay), and stability of TMZ, compared with the ready-to-use Ped-TMZ suspension. Pediatric patients who require TMZ treatment typically receive doses of 80–120 mg [15]. For the purpose of this study, a dose of 90 mg was chosen corresponding to a child under the age of 6 exhibiting a BSA of 0.6 m^2^ and treated with 150 mg/m^2^ of TMZ. Different liquids and soft foods were selected with regard to the reported use [20] or likelihood to be used in the targeted pediatric population (children from the age of 1 year) and to cover the range of various food components (e.g., caloric content), physicochemical properties (e.g., pH, texture) and temperature administration (e.g., ambient or hot). Due to the pH-dependent hydrolysis of TMZ [21], vehicles of various pH levels were selected. Apple sauce and apple juice were selected for their acidic pH environment. The other vehicles (chocolate cream, mashed, and infant milk) were chosen for their anticipated neutral pH and their different textures, compositions, and temperature administrations. Additionally, apple sauce and apple juice (in which the chemical stability of TMZ is supposed to be favorable) were used to assess the potential impact of the vehicle texture (liquid vs. soft food) on the delivered dose, e.g., related to any potential loss of the vehicle. The holding time was assessed over one hour after the preparation of the mixture and was considered a reasonable timeframe consistent with real life.

## 2. Materials and Methods

### 2.1. Dosage Forms

In total, 20 mg capsules and 5 mg capsules of Temodal^®^ were purchased from Merck Sharp & Dohme, Puteaux, France.

Ped-TZM (KIZFIZO^®^, ORPHELIA Pharma, Paris, France), a ready-to-use suspension containing 40 mg/mL of TMZ, is an investigational product comprising the following excipients: xanthan gum; citric acid, silicon dioxide, sodium benzoate, sucralose, cola flavor, and purified water. Ped-TMZ is manufactured by mixing excipients in purified water before the introduction of TMZ. After at least 30 min of stirring with TMZ, the suspension is filled into bottles [22].

### 2.2. Liquid and Soft Food Vehicles

Liquid and food vehicles are listed in Table 1. They were purchased from a local supermarket in France. The pH was measured at ambient temperature using the SevenGo Duo Pro pH meter (Mettler-Toledo SAS, Viroflay, France). The nutrition information of the vehicles is reported in Table 2.

### 2.3. Accuracy of the Delivered Dose Study (TMZ Assay)

Ninety (90) mg of TMZ powder was prepared from four Temodal^®^ capsules of 20 mg and two capsules of 5 mg and compared to 88 mg (2.2 mL) of Ped-TMZ.

The TMZ capsules were opened, and their contents were mixed with either a soft or liquid vehicle: 100 g apple sauce or 100 mL apple juice. The apple sauce/juice preparations were mixed or agitated in the original container with a spoon for 30 s. Analytical processing was then carried out according to the sample preparation adapted from the analytical testing applied for Ped-TMZ in order to reduce the food matrix effect. The food/TMZ mixture was transferred using the same spoon as for mixing into a 2000 mL volumetric flask containing 500 mL of dilution solvent (DS) (0.5% *v*/*v* of aqueous solution of glacial acetic acid, J.T. Baker, HPLC Grade) for analytical processing. Each flask was magnetically stirred for 10 min, and the volume was increased to 2000 mL with DS. After magnetically stirring for 10 min, the solution was centrifuged for 10 min at 10,000 rpm. The supernatant was assayed by HPLC-UV to determine the percentage of TMZ recovered from the capsule-derived mixture.

The Ped-TMZ suspension was sampled using the provided co-packaged device (5 mL syringe with 0.1 mL precision) and transferred directly into a 500 mL volumetric flask containing 250 mL of DS for analytical processing. Each flask was magnetically stirred for 10 min and completed to 500 mL with DS. After an additional 10 min of stirring, 5 mL of the Ped-TMZ sample solution was diluted into 20 mL of DS and centrifuged for 10 min at 10,000 rpm. The supernatant was assayed using HPLC-UV. Temozolomide quantities in the apple sauce/juice preparations (90 mg) were compared with 2.2 mL of ready-to-use Ped-TMZ 40 mg/mL oral suspension, equivalent to 88 mg TMZ. The recovery of TMZ was calculated as the ratio of the delivered dose/theoretical dose (where the theoretical dose is 90 mg for Temodal and 88 mg for Ped-TMZ). Values were also assessed against the predefined acceptance criteria for TMZ assay (95.0–105.0%).

All experiments were replicated three times. They involved six non-analyst operators, not particularly well trained or educated in pharmaceutical practice, acting as caregivers or parents in the current study. These non-analyst operators did the mixing step, transferred the content into the flask for the Temodal manipulation, and did the sampling for Ped-TMZ. For safety reasons, analytical technicians opened and emptied the Temodal capsules into the liquid and soft food and performed the analytical sample preparation and assay.

### 2.4. Stability of TMZ Study

Capsule contents (90 mg of TMZ) were mixed with different children’s food vehicles: in the original container for apple sauce (100 g) and chocolate cream (125 g), in 100 g of mashed potatoes, and in 100 mL of infant milk. TMZ powder was left in contact with the food vehicles for varying periods of time (0, 30, and 60 min), then transferred into a volumetric flask containing DS and centrifuged for TMZ and amino-imidazole-carboxamide (AIC) quantification according to the operating conditions previously described. All experiments were replicated three times for each tested matrix. They were performed by analytical, trained technicians.

For the Ped-TMZ, the syringes were filled with 2 mL of Ped-TMZ and stored flat for 24 h at 25 °C/60% RH and 30 °C/65% RH. The content of the syringe was then transferred into a volumetric flask containing DS and centrifuged for TMZ and amino-imidazole-carboxamide (AIC) quantification according to the operating conditions previously described. This stability study was performed on two filled syringes for each time point. It was carried out by analytical, trained technicians.

### 2.5. Samples Analysis

The operating conditions were similar between the TMZ assay and degradation products. TMZ and its main degradation product, AIC, were assayed using ultraviolet high-performance liquid chromatography (HPLC-UV) (Agilent HP1260, Agilent Technologies Inc., Santa Clara, CA, USA) equipped with a UV detector. A Luna C18 column (150 × 4.6 µm, 5 µm) was utilized at the temperature of 30 °C. The injection volume of the sample was 20 µL. The mobile phase A was composed of a mixture of methanol (gradient grade) (40 mL) with a 0.5% (*v*/*v*) aqueous solution of glacial acetic acid R (HPLC grade) (960 mL) in which 0.94 g of sodium hexane sulfonate (HPLC grade) was dissolved. The mobile phase B was composed of methanol (gradient grade). The selected elution gradient started with a 100% mobile phase A, then a 45%/55% phase mobile A/phase mobile B, and again a 100% mobile phase A. TMZ and its degradation product were detected at 254 nm. The method was validated according to ICH Q2 [23]. For the TMZ assay, the method is linear over the range of 70% to 130% of the theoretical content of TMZ in the drug product (R^2^ = 1.0000). The accuracy and precision were tested on the three following series: 70%, 100%, and 130% of TMZ. The mean recovery ranged from 99.8% to 99.9%, and all individual recoveries complied with the 98.0–102.0% acceptance criteria. For precision, the coefficient of variation ranged from 0.2% to 0.4% for repeatability and from 0.4% to 0.5% for intermediate precision. The specificity of the method between TMZ and its degradation products was observed. The linearity over the range of 0.05% to 8.0% of the theoretical content of TMZ in the drug product (R^2^ = 1.000) was compliant with the control of TMZ degradation products. The accuracy and precision were tested on the five following series: 0.05%, 0.2%, 2.5%, 5.0%, and 8.0% of TMZ. The mean recovery ranged from 99.6% to 100.9%, and all individual recovery complied with the 75.0–125.0% and 90.0–110.0% acceptance criteria for the LOQ and 2xLOQ levels and other levels, respectively. For precision, the coefficient of variation ranged from 0.4% to 1.2% for repeatability and from 0.8% to 1.4% for intermediate precision. The stability of the solution was demonstrated for the TMZ and degradation product assays.

The method suitability was also verified in the presence of a food vehicle. Specificity was verified for TMZ and AIC in the presence of a food vehicle. Recovery ranged between 98.51% and 99.70%, whatever the vehicle. The linearity was demonstrated from 20% to 120% of the theoretical content of TMZ. Acceptance criteria were predefined according to the drug product performance of Ped-TMZ for TMZ (95.0–105.0%) and AIC (<1%) content.

### 2.6. Statistical Analysis

To determine the impact of the food vehicles on the amounts of TMZ and AIC in the samples, statistical analyses (JMP software used, version 16) were performed. The effect of the contact time with soft food was assessed in the stability study. The impact of the operator and repetition on TMZ content was also evaluated in both studies. One-way ANOVA or a mixed model was used to compare the dose recovery or AIC content. A Levene test was used for the comparison of variances.

## 3. Results

### 3.1. Accuracy of the Delivered Dose

**Figure 1:**
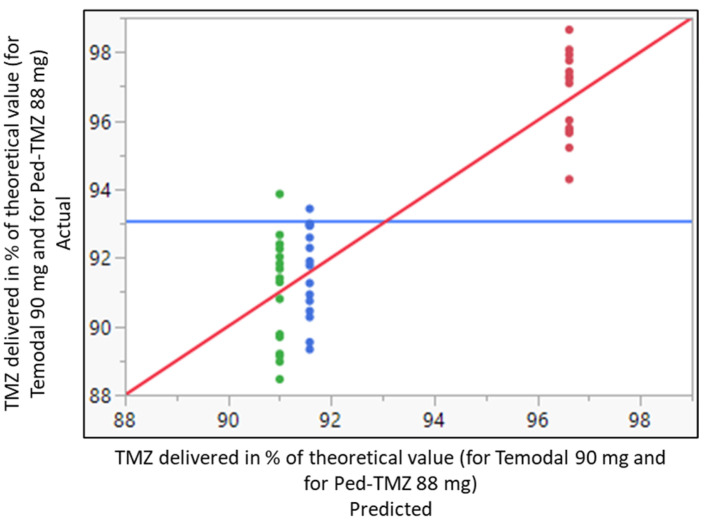
Whole model actual by predicted plot for the delivered dose of TMZ in % of the theoretical value (90 mg for Temodal and 88 mg for Ped-TMZ) after Temodal manipulation (● Temodal + apple juice; ● Temodal + apple sauce) or using ready-to-use Ped-TMZ (● Ped-TMZ). Predicted RMSE = 1.3778 Rsq = 0.78 *p*-value < 0.0001. Blue horizontal line represents the average of the delivered dose. Red diagonal line represents the line of fit for the model actual vs. predicted value. The colors are the 3 levels of the predicted delivered doses.

Immediately after dispensing the TMZ capsules into apple juice and apple sauce, both drug manipulations presented a similar distribution of TMZ content (Figure 1). The mean ± SD TMZ recovery was 91.0 ± 1.5% and 91.6 ± 1.4%, using apple juice and apple sauce, respectively, without any significant statistical differences between both vehicles (confidence interval −1.6939/0.53059). The delivered doses consistently fell below the predefined acceptance range (95.0–105.0%). In comparison, the recovery of TMZ in the Ped-TMZ suspension was significantly higher than that observed after handling the TMZ capsules (96.6 ± 1.2%; *p*-value _ANOVA_ < 0.0001), with TMZ quantities falling within predefined specifications (Figure 1). The main degradation product (AIC) was additionally controlled, and the results were found below the limit of quantification (LOQ = 0.1%) in all samples of Temodal mixed into apple juice or apple sauce.

The ready-to-use Ped-TMZ liquid formulation was statistically different from the TMZ recovered after handling capsules with regard to the mean of the response. The liquid formulation systematically increased the percentage of the delivered dose by 3.55 points in comparison with Temodal mixed with apple sauce (estimate calculated from the linear mixed model using REML (restricted maximum likelihood) approach).

The findings were reproducible across all six operators for the three groups, as shown in the variance comparison in Table 3. The variability was equivalent regardless of the group (Ped-TMZ, Temodal + apple juice, Temodal + apple juice; Levene test *p*-value = 0.8648).

**Figure 2:**
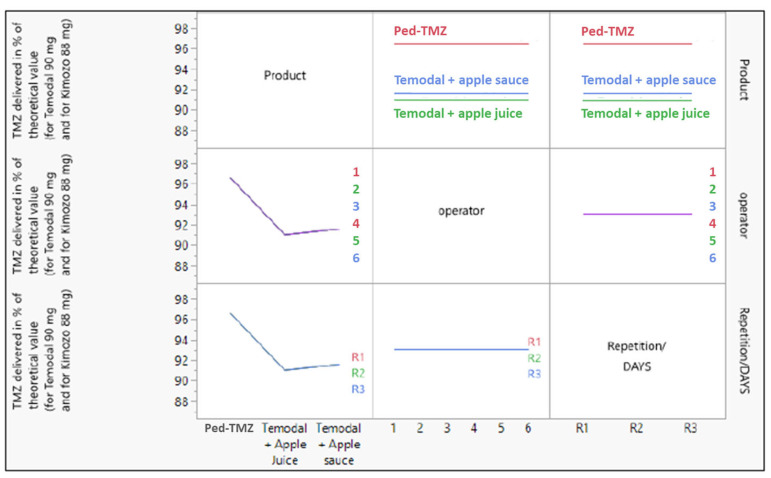
Effect of the three variables (product (Ped-TMZ, Temodal + Apple Juice, Temodal + Apple Sauce), operator (1 to 6), and repetition (R1, R2, R3)) on the response (TMZ delivered in % of the theoretical value).

The analysis was completed with the determination of the source of variance using a linear model testing the three factors: (i) the product (Ped-TMZ, TMZ capsule mixed with apple juice, and TMZ capsule mixed with apple sauce), (ii) the operator (*n* = 6) and (iii) the repetition (*n* = 3). The product was the only source of variance in the accuracy experiments; operator or repetition did not affect the TMZ recovery (Figure 2).

### 3.2. Chemical and Physical Stability

**Figure 3:**
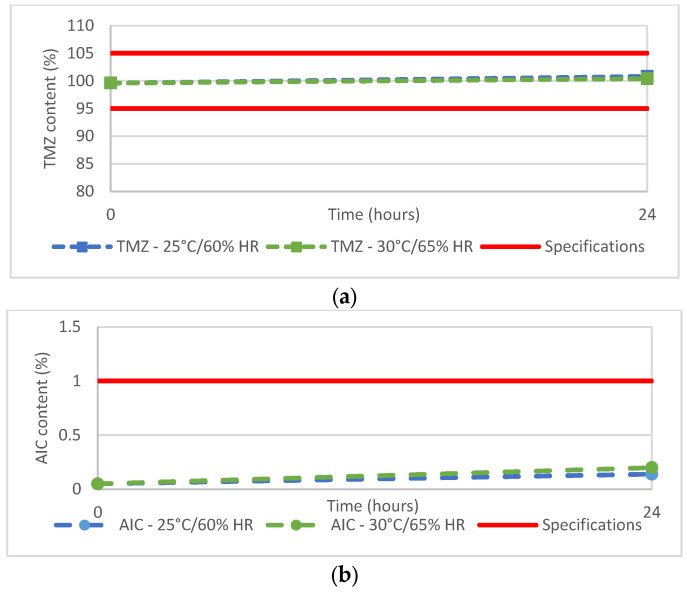
(**a**) TMZ and (**b**) AIC content of Ped-TMZ after 24 h storage at 25 °C or 30 °C.

Although the ready-to-use Ped-TMZ product is to be stored at 2–8 °C, the stability of the suspension stored in the oral syringe was investigated. The TMZ content was 100.8% or 100.4% when syringes were kept at 25 °C/60% RH or 30 °C/65% RH, respectively, for 24 h, a duration far exceeding requirements as syringes are to be prepared extemporaneously (i.e., just before administration of the drug product). This study demonstrated that the TMZ (Figure 3a) content remained within the predefined acceptance criteria (95%–105%). In addition, very limited degradation was noticed with a slight increase in the AIC from 0.05% to 0.14% or 0.2% (Figure 3b) after 24 h of storage at 25 °C/60% RH or 30 °C/65% RH, respectively. These levels are well below the predefined specification (<1% for AIC).

**Figure 4:**
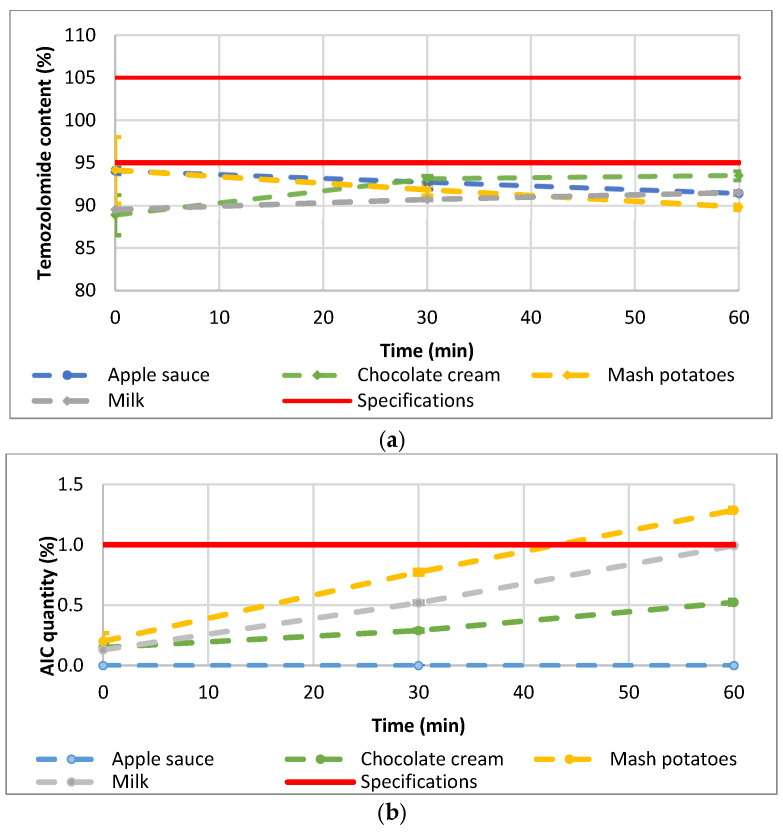
(**a**) mean TMZ and (**b**) mean AIC content after mixing the TMZ capsule contents with food (*n* = 3). Error bars represent SD (range for TMZ: 0.09–3.88; range for AIC: 0.01–0.07).

Thirty-six (36) samples were prepared by mixing TMZ capsule contents with four different soft food vehicles (apple sauce, chocolate cream, infant milk, and mashed potatoes). The mean TMZ content for the 36 preparations is reported by vehicle over time in Figure 4a. The TMZ content from the mashed potatoes or apple sauce decreased from 94.2 ± 3.9% and 94.1 ± 0.4% to 89.8 ± 0.3% and 91.4 ± 0.1%, respectively, after 60 min of contact with food. TMZ from the milk mixture remained stable with content between 89.5 ± 0.2 (T0) and 91.5 ± 0.2% (60 min). TMZ content from the chocolate cream fluctuated from 88.9 ± 2.4% to 93.5 ± 0.5%. However, whatever the vehicle (apple sauce, chocolate cream, mashed potatoes, or milk) or the time point (0, 30 min, 60 min), the TMZ content was systematically below the predefined 95% lower limit except for one sample at T0. The mean AIC content over time is shown in Figure 4b, with evidence of TMZ degradation with up to 0.5%, 1.0%, and 1.3% of AIC quantified in the mixture of Temodal with chocolate cream, milk, and mashed potatoes, respectively, after 60 min of contact with food. Only the apple sauce prevented any degradation of TMZ into AIC (less than LOQ 0.1%).

**Figure 5:**
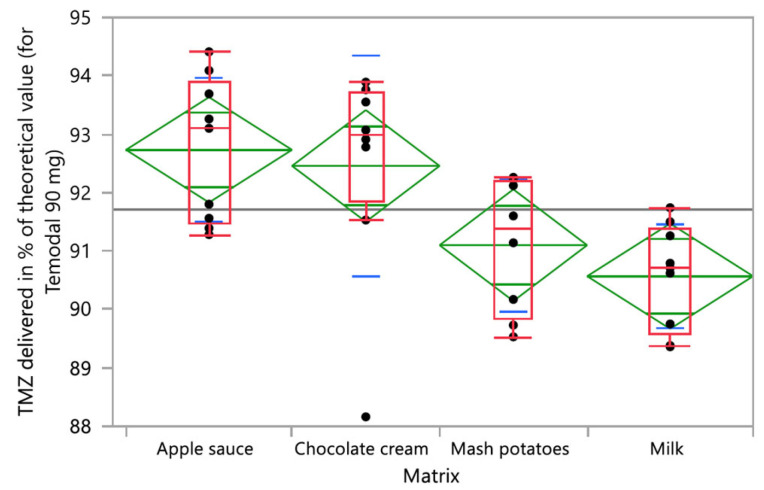
One-way analysis of TMZ delivered in % of the theoretical value (90 mg for Temodal) by vehicle (matrix). Box plot (median, 25th and 75th percentiles, min and max corresponding to 1.5 × IQR (Inter Quartile Range) in red, mean with confidence interval in green, and and lines that are at one standard deviation of the means in blue.

The impact of the different studied factors (vehicle, time, and operator) was analyzed based on a one-factor ANOVA or Kruskal–Wallis test according to normal distribution or not (test for homogeneity of variance). The four tested food vehicles (apple sauce, chocolate cream, infant milk, and mashed potatoes) had a significant impact on the TMZ delivered dose (*p*-value _ANOVA_ = 0.0042; Figure 5), whilst the vehicle factor did not have any significant effect on the degradation product AIC (*p*-value _Kruskal-Wallis_ = 0.1850). One-factor analysis has been used here for illustration purposes.

**Figure 6:**
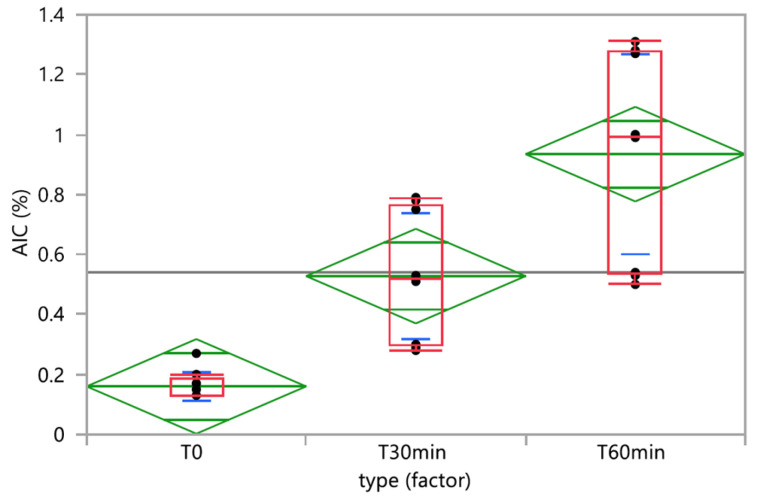
One-way analysis of AIC (%) by time. Box plot (median, 25th and 75th percentiles, min and max corresponding to 1.5 × IQR (Inter Quartile Range) in red, means with 95% confidence interval in green, and lines that are at one standard deviation of the means in blue.

Time did not have a significant effect on the TMZ-delivered dose (*p*-value _ANOVA_ = 0.5655). Nevertheless, one vehicle (milk) had a significant time effect on the TMZ dose, and two vehicles (milk and chocolate cream) evidenced homogeneity and physical stability issues with initial TMZ dosing below 90% and fluctuating over time (Figure 4a). The quantities of AIC, the main degradation product of TMZ, significantly increased over time when the TMZ capsules were mixed with vehicles (*p*-value _Kruskall-Wallis_ < 0.0001) (Figure 6). In the one-way analysis, the food vehicle factor did not impact the AIC content (*p*-value _Kruskall-Wallis_ = 0.1850). Nevertheless, the analysis of the individual food effect over time showed that AIC content increased significantly (*p*-value < 0.0001) when TMZ capsules were mixed with chocolate cream, milk, and mashed potatoes, the three vehicles for which pH is between 6 and 7. The AIC quantity was highest when the capsules were mixed with mashed potatoes, exceeding the predefined AIC content acceptance criteria (<1%) 60 min after mixing.

**Figure 7:**
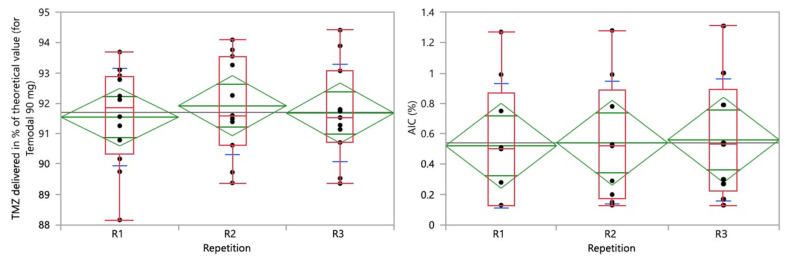
One-way analysis of TMZ delivered in % of the theoretical value (90 mg for Temodal) (**left**) and AIC (%) (**right**) by repetition. Box plot (median, 25th and 75th percentiles, min and max corresponding to 1.5 × IQR (Inter Quartile Range) in red, means with 95% confidence interval in green, and lines that are at one standard deviation of the means in blue.

The effect of repetition was analyzed on both the TMZ delivered and AIC results. The one-way analysis is presented in Figure 7, demonstrating the homogeneity of the variance (*p* = 0.8519 and *p* = 0.7537 for TMZ and AIC, respectively).

Combining all the assays performed, 72 samples were prepared from capsule contents mixed with 5 liquid or soft food vehicles, among which only 1 sample (1.4%) (1 out of 3 replicates using mashed potatoes at T0) delivered the right dose of TMZ (95.0%–105.0%). This means that 98.6% of samples prepared from the mixture of capsules with food would have led to underdosing of the patient.

## 4. Discussion

TMZ plays a crucial role in the chemotherapy treatment of pediatric oncology conditions, including recurrent or refractory gliomas [1,2], neuroblastoma [3,4,5], medulloblastoma [6], and rhabdomyosarcoma [7]. Despite its importance in the treatment of cancers affecting very young patients, there is currently no suitable formulation adapted for them.

When lacking appropriate pediatric forms, drug manipulations are common practices in both outpatient and inpatient settings [16]. Parents and caregivers use drug manipulation to achieve a better taste or to easily adjust the dose, whereas nurses perform it to reduce the size of the dosage forms or to facilitate administration through a feeding tube. In any case, the lack of information in SmPC or patient information leaflets or incomplete advice from their pharmacy often leads the parents, caregivers, or nurses to improvise, possibly using incompatible vehicles [24]. The impact of mixing capsules, crushed tablets, and other oral compounded formulations with foods and beverages has been evaluated in some studies [25,26,27,28]. It was highlighted that the physicochemical properties of food vehicles are an important consideration for drug exposure [29].

For TMZ administration, caregivers open capsules and mix their contents with any kind of beverage or soft foods. This practice poses multiple risks, including exposure of the caregiver to a cytotoxic drug, imprecise delivery and dosing, partial drug intake if the food or beverage is not totally ingested or consumed, and uncontrolled stability of the active compound [8,15,20]. Here, we investigated the dosing accuracy and drug stability of TMZ in various food vehicles commonly used to aid administration to pediatric patients [18,29] and whether an investigational oral suspension of TMZ could offer improved dosage accuracy and stability.

When TMZ capsules were mixed with apple sauce and apple juice, the delivered dose of TMZ consistently fell short of predefined specifications, suggesting the loss of TMZ during the preparation process, whatever the texture of the vehicle (liquid or soft food). Since the pH was acid for both vehicles (3.5–3.8), the experimental conditions were chemically favorable for TMZ stability (confirmed in the stability part of the study with apple sauce), suggesting that the loss of TMZ content may occur during the preparation. The process of twisting apart and separating the two parts of the capsule requires care and dexterity, and loss of powder onto gloves, surfaces, or utensils may occur. Some powder may also remain lodged inside the capsule, meaning that the full required dose is not dispensed into the food. This decrease in the delivered dose is likely to be underestimated in our study as the capsules were opened and emptied by skilled laboratory technicians for safety reasons. Loss may also occur after mixing the TMZ powder with the vehicle during the transfer with the spoon or syringe, mimicking administration to the child since the container was not totally emptied and/or rinsed. Interestingly, our study showed a good intra and inter-operator reproducibility. For non-analyst operators, who were not specifically well trained or educated, this minimal variability in their practice contrasts with the significant difference between nurses and hospital pharmacists in the comparative assessment highlighted by Nguyen et al. [30].

Mixing TMZ capsules with food also significantly affected the chemical stability of TMZ in a time-dependent manner. The food or drink vehicles had a significant impact on TMZ quantity, and the amount of degradation product AIC significantly increased in three out of the four food vehicles over the 60 min testing period. Mashed potato showed the most apparent degradation, while apple sauce exhibited minimal degradation. These findings are supported by a previous study involving 15 fluids, soft foods, and suspension vehicles, which indicated that drug bioavailability can be significantly influenced by various physicochemical properties of the vehicle, including pH, surface tension, and viscosity [31]. In the current study, differences in the AIC content over time may be essentially attributed to the pH of the food vehicles. Unlike apple sauce, which has an acidic pH (typically between 3.3 and 4.6) [32], the other three food vehicles had pH values ranging from 6.4 to 6.8. Since TMZ starts degrading at pH 7 and above, the increase in AIC quantity over time can be attributed to the neutral pH of the food. The fast degradation of TMZ in food presents practical challenges for parents and caregivers, as each dose needs to be freshly prepared and consumed promptly and fully to ensure proper dosage. This can be particularly challenging for children with reduced appetite or those who find the taste unpalatable, as is the case for TMZ, which exhibits a bitter taste.

Altogether, an important teaching of our study is that, despite the sample preparation being partially above the real-life standards (i.e., by skilled technicians) and assuming the complete consumption of the preparation by the child, 99% of the capsule-food/drink mixes would not have delivered the proper dose of TMZ (i.e., <95% of the targeted dose) including one sample with TMZ content as low as 86.9%, which is not acceptable for an anticancer medicine. Considering the cumulative risks associated with medicine manipulation, i.e., capsule opening and mixing by the parents, the difficulties in having a complete intake of the mixture by the child, and the time needed for this preparation that could also increase the risk of errors, the delivered dose may be dramatically reduced.

Another factor that may further contribute to TMZ underexposure is the impact of food co-administration on drug absorption. In a phase I dose-escalation and pharmacokinetic (PK) study of TMZ where 15 adult patients suffering from refractory or relapsing malignancies were enrolled, there was a 9% reduction in the AUC_0–24_ and a 33% reduction in C_max_ in fed patients compared with the fasted group [1]. In the pediatric population, due to differences in physiology, anatomy, and the composition of food consumed, the food–drug interaction cannot be predicted based on adult studies [33]. That raises additional uncertainty on drug exposure when medicines are co-administered with food to children. The impact on TMZ bioavailability of co-administering 100–125 g of food, as per clinical practice or these experiments, is not known but may not be null.

Liquid formulations for oral administration are particularly crucial in pediatric patient treatment since they allow dose adjustment to weight or BSA, flexibility in administration by swallowing or feeding tube, and ease of use, and they significantly reduce the risk of manipulation [11]. Nevertheless, in the course of their development, these formulations need to address the palatability that may be an issue [11] and the patient acceptability in general [14], for which the dose volume is a major consideration [9]. The formulation of oral liquid products may also be challenging, as they generally require the use of excipients that are potentially harmful, e.g., antimicrobial preservatives for multidose containers, sweeteners, or flavoring agents [34,35]. They may also represent larger storage/transport volumes than solid dosage forms [36]. A proof-of-concept study demonstrated that preparation of a liquid formulation of TMZ from the intravenous infusion powder was feasible but it exhibited modest stability (13 weeks at 5 °C) and very low concentration (1.25 mg/mL), thereby requiring large administration volumes (e.g., 72 mL for 90 mg as in our study), not suitable for a pediatric oral formulation [8] in comparison to the recommended dose volumes for pediatric liquid products (less than 5 mL for children under 5 years and less than 10 mL for children of 5 years and older) [9]. Subsequently, the Gustave Roussy Cancer Campus developed a hospital-compounded oral suspension prepared from TMZ capsules and selected excipients, which exhibited satisfactory chemical and physical stability as well as lower distribution volumes [15]. This prototype was further developed at an industrial scale and led to the development of Ped-TMZ, with further improved long-term stability and higher TMZ concentration, allowing the administration of small volumes in line with EMA recommendations [9].

The efficacy, safety, and stability of Ped-TMZ are being tested across a range of studies. An open-label, randomized, multicenter study in patients with primary central nervous system (NCT04467346) showed the bioequivalence between Ped-TMZ and TMZ capsules [37]. An international, open-label, non-randomized, prospective, single-arm phase I study is underway to determine the PK, acceptability, and safety of Ped-TMZ in the pediatric population (NCT04610736).

## 5. Conclusions

TMZ dispensed with food using a spoon is systematically underdosed, as evidenced by poor recovery and instability. Our experiments suggest that mixing TMZ capsule contents with food may result in significant underexposure in pediatric patients. The ready-to-use and taste-masked oral suspension Ped-TMZ offers superior dosing accuracy and better stability than TMZ vehicle mixtures. Ped-TMZ addresses the critical and long-standing unmet medical need for young cancer patients with an age-appropriate formulation of TMZ, which was underlined by the EMA as early as 2014 [19].

## Figures and Tables

**Table 1:** Vehicles investigated in the study.

Liquid/Soft Food	Brand Name	Packaging	pH	Texture
Apple juice	Joker	1 L bottle	3.5	Liquid
Apple sauce	Andros	100 g pot	3.8	Soft food
Chocolate cream	Danette	125 g pot	6.8	Soft food
Infant milk *	Candia baby	250 mL bottle	6.8	Liquid
Mash potatoes	Mousseline	130 g sachet	6.4	Soft food

* standard liquid infant milk formula based on cow’s milk, containing mineral/vitamin supplements.

**Table 2:** Nutrition facts of the investigated vehicles (given for 100 mL or 100 g).

	Apple Juice	Apple Sauce	Chocolate Cream	Infant Milk	Mash Potatoes
Calories (kJ)	187	296	516	261	280
Calories (kcal)	44	70	122	62	67
Fat (g)	0	0.2	3.0	3.2	0.8
Carbohydrates (g)	10	16.0	20.0	7.0	2.5
Proteins (g)	0	0.3	3.3	1.4	2.4

**Table 3:** Statistical analysis of the variability of the three groups: Ped-TMZ, Temodal mixed with apple juice, and Temodal mixed with apple sauce.

Group	Standard Deviation	Median Absolution Deviation (Robust Deviation Measure)
Ped-TMZ	1.246784	1.104444
Temodal + apple juice	1.488973	1.156667
Temodal + apple sauce	1.356263	1.155000

## Data Availability

Due to commercial restrictions, the data is not publicly available. Data are contained within the article.
